# Evaluation of Real-Time Quaking-Induced Conversion, ELISA, and Immunohistochemistry for Chronic Wasting Disease Diagnosis

**DOI:** 10.3389/fvets.2021.824815

**Published:** 2022-01-18

**Authors:** Carine L. Holz, Joseph R. Darish, Kelly Straka, Nicole Grosjean, Steven Bolin, Matti Kiupel, Srinand Sreevatsan

**Affiliations:** ^1^Department of Pathobiology and Diagnostic Investigation, College of Veterinary Medicine, Michigan State University, East Lansing, MI, United States; ^2^Michigan Department of Natural Resources, Lansing, MI, United States

**Keywords:** deer, prions, RT-QuIC, immunohistochemistry, diagnostics, chronic wasting disease (CWD)

## Abstract

Chronic wasting disease (CWD) is a transmissible prion disorder, primarily affecting free-ranging and captive cervids in North America (United States and Canada), South Korea, and Europe (Finland, Norway, and Sweden). Current diagnostic methods used in the United States for detection of CWD in hunter harvested deer involve demonstration of the causal misfolded prion protein (PrP^CWD^) in the obex or retropharyngeal lymph nodes (RLNs) using an antigen detection ELISA as a screening tool, followed by a confirmation by the gold standard method, immunohistochemistry (IHC). Real-time quaking-induced conversion (RT-QuIC) assay is a newer approach that amplifies misfolded CWD prions *in vitro* and has facilitated CWD prion detection in a variety of tissues, body fluids, and excreta. The current study was undertaken to compare ELISA, IHC, and RT-QuIC on RLNs (*n* = 1,300 animals) from white-tailed deer (WTD) in Michigan. In addition, prescapular, prefemoral and popliteal lymph nodes collected from a small subset (*n* = 7) of animals were tested. Lastly, the location of the positive samples within Michigan was documented and the percentage of CWD positive RLNs was calculated by sex and age. ELISA and RT-QuIC detected PrP^CWD^ in 184 and 178 out of 1,300 RLNs, respectively. Of the 184 ELISA positive samples, 176 were also IHC positive for CWD. There were seven discordant results when comparing IHC and ELISA. RT-QuIC revealed that six of the seven samples matched the IHC outcomes. One RLN was negative by IHC, but positive by ELISA and RT-QuIC. RT-QuIC, IHC, and ELISA also detected PrP^CWD^ in prescapular, prefemoral and popliteal lymph nodes. CWD infection heterogeneities were observed in different age and sex groups, with young males having higher CWD prevalence. All, except one, CWD positive RLNs analyzed were from ten Counties geographically located in the West Michigan region of the Lower Peninsula. Taken together, we show evidence that the RT-QuIC assay is comparable to ELISA and IHC and could be helpful for routine CWD detection in surveillance programs. RT-QuIC also demonstrated that CWD prions are distributed across lymph nodes in a variety of anatomic locations. A multi-laboratory validation on blinded sample panels is underway and is likely to help to provide insight into the variability (lab-to-lab), analytical sensitivity, and specificity of gold standard diagnostics vs. RT-QuIC assay.

## Introduction

Chronic wasting disease (CWD) is the only known transmissible spongiform encephalopathy to occur in free-ranging wildlife populations, naturally infecting elk, moose, and various deer species. To date, the disease has been reported in 26 American states, four Canadian provinces, South Korea, Norway, Finland, and Sweden (1–5). It continues to spread across North America through new and ongoing outbreaks. The exact mechanisms of CWD spread remains unclear. Direct contact with saliva, urine, feces, or aerosols from infected animals, as well as indirect (environmental) contact through the ingestion of infectious prions bound to soil or plants, and vertical transmission, likely contribute to disease spread (6–8). Mathematical models of CWD prevalence and dynamics support the hypothesis that direct transmission accounts for most transmission events, with both population density and contact frequency contributing to CWD spread (9, 10). However, the extraordinary stability and resistance to degradation of the CWD prion allows it to remain infectious in the environment for several years (6, 11). Furthermore, 18 to 20 months may pass between initial infection and the onset of clinical signs. The long incubation period contributes to CWD transmission as asymptomatic infected animals can substantially contaminate the environment through body secretions such as urine, saliva, and feces (12–14). However, animals in the first stages of the disease contain concentration of prions in their tissues that cannot be detected by standard methods such as ELISA or immunohistochemistry (IHC).

As a prion disease, CWD is caused by a pathogenic, misfolded conformation of the normal, natively folded, cellular prion protein to a pathogenic prion conformer PrP^CWD^, which accumulates mostly in the central nervous system (15–18). During the infection, PrP^CWD^ propagates via a process of seeded polymerization, characterized by increased formation of β-sheets, propensity to aggregate into amyloid fibrils, and resistance to protease and acid digestion (16, 19–21). Definitive diagnosis of CWD in the United States relies on the detection of PrP^CWD^ in the brain or retropharyngeal lymph nodes (RLNs) by federally recognized diagnostic methods performed at approved laboratories: ELISA and IHC. Despite their reliability, such methods have limited sensitivity and application across various tissues and body excreta in comparison to *in vitro* amplification methods for prion detection, such as protein misfolding cyclic amplification and real-time quaking-induced conversion (RT-QuIC) assays. RT-QuIC assay offers a newer, high-throughput approach that can detect minute amounts of prions in tissues and secretions of CWD-infected animals with the additional advantage of avoiding the proteolytic or acidic pretreatments commonly required for the conventional CWD detection assays (ELISA and IHC). Even though RT-QuIC has been reported to efficiently amplify PrP^CWD^ in a variety of tissues (e.g., RLNs, brain, ear pinna, third eyelid, muscle), body fluids (e.g., nasal swabs, CSF, blood), and excreta (e.g., saliva, urine, feces) of animals at clinical and, sometimes, at preclinical stages of disease, the method is not yet approved for official CWD surveillance programs (13, 22–30). Nevertheless, sensitive and rapid detection of low levels of prions in early stages of infection would aid substantially in understanding the epidemiology of the disease and enable assessment of mitigation based on management strategies to minimize disease transmission.

A few studies have investigated the utility of RT-QuIC in comparison to current immunodetection assays on free-ranging cervids (23, 28, 30). In the present study, the consistency of results between RT-QuIC, ELISA and IHC for the detection of CWD was analyzed in 1,300 RLNs of white-tailed deer (WTD, *Odocoileus virginianus*) collected between 2015 and 2021 in Michigan. Furthermore, the utility of RT-QuIC assay for the detection of CWD prions in a broader set of lymphatic tissues was examined. Lastly, the prevalence of CWD by age and sex was calculated and the location of the positive samples within Michigan documented. Our results demonstrate that males present two times higher prevalence than female WTD in Michigan. CWD infection seems to increase with age, but steadily declines after the fourth year of life. Our data also suggests that CWD is broadly present in lymph nodes of infected deer. Moreover, the RT-QuIC assay performance is comparable to conventional assays for CWD detection in lymph nodes of WTD in terms of sensitivity and it could be helpful as a new CWD diagnostic tool.

## Materials and Methods

### Sample Collection

**Figure 1:**
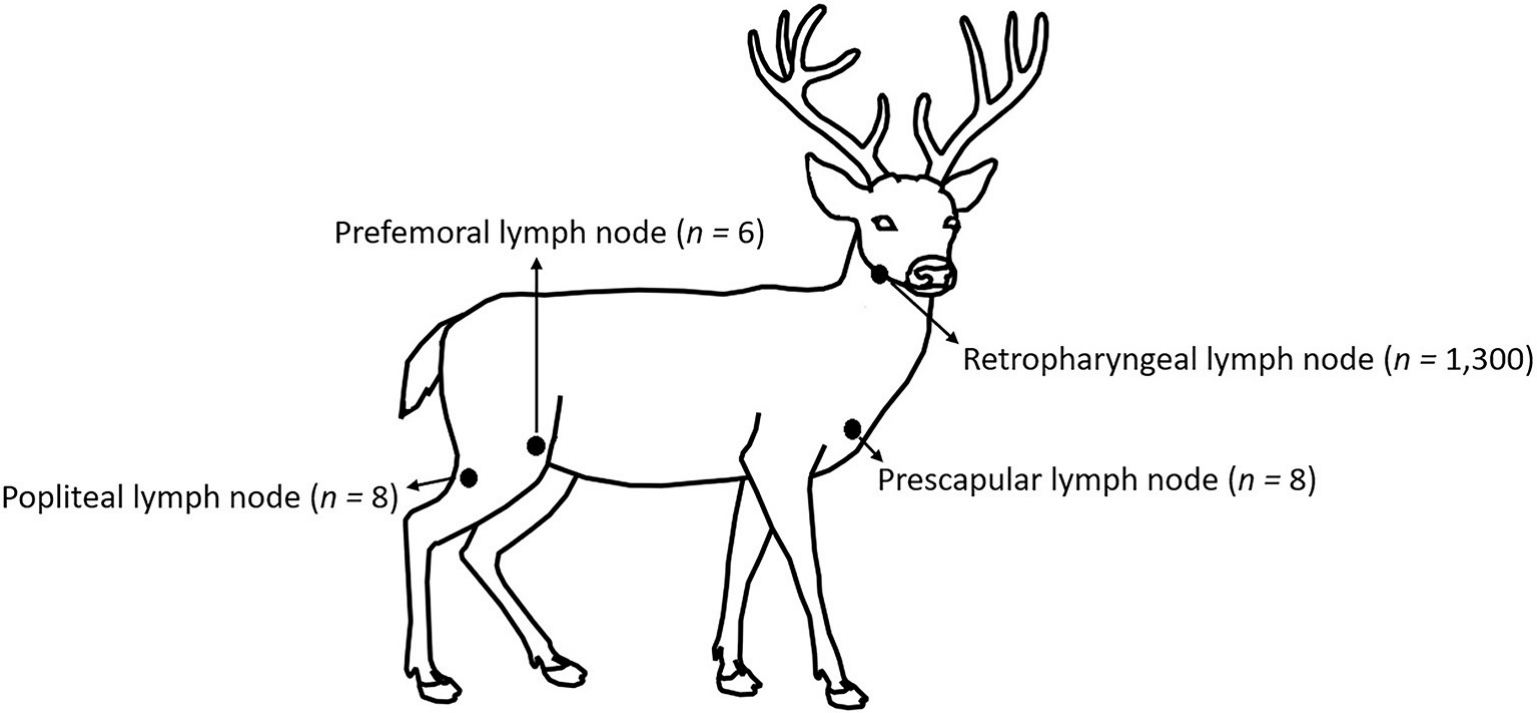
Location and number of lymph nodes tested in this study.

No animal-use-protocol approval was required for this study since all samples were procured from deer submitted for testing at the Michigan State University, Veterinary Diagnostic Laboratory (MSU-VDL). Anatomic location and number of lymph nodes tested on this work are shown in Figure 1. One thousand three hundred WTD RLNs, eight prescapular lymph nodes, eight popliteal lymph nodes, and six prefemoral lymph nodes were collected post-mortem by trained laboratory personnel from 2015 to 2021 as part of the CWD surveillance in Michigan. Portions of lymph node were stored at−20°C (for ELISA and RT-QuIC), while the remaining node was fixed in 10% neutral-buffered formalin (for IHC). Information about the location, sex, and age (estimation based on tooth replacement and wear) of the animals was also collected.

### ELISA

The TeSeE™ Short Assay Protocol (SAP) Combi Kit (Bio-Rad) ELISA was performed following the manufacturer's instructions. Briefly, 2 or 3 wedge shaped sections of lymph node cortex with a total weight of 180 to 220 mg were placed into a grinding tube with lysis buffer and a large ceramic bead, all supplied in the SAP Combi Kit. The pieces of lymph node were homogenized using a FastPrep® (MP Biomedicals, Irvine, CA) bead-beater homogenizer for 2 cycles of 45 s at 6.5 m/sec. Next, 250 μl of homogenate from the grinding tube was placed into a 2 ml microcentrifuge tube, mixed with 250 μl of a proteinase K solution supplied in the Kit, and incubated at 35 to 39°C for 10 min. Then 250 μl of reagent B from the SAP Combi Kit was added to the microcentrifuge tube and mixed by inversion. Finally, the mixture was clarified by centrifugation for 7 min at 15,000 rcf at 20°C. The supernatant was removed, the pelleted material was air dried, and 25 μl of reagent C from the kit was added to the microcentrifuge tube. After a 5-min incubation at 95–105°C, the contents of the tube were cooled and then briefly vortexed. Except for the homogenization step, this process was repeated in duplicate for each sample that was non-negative in the ensuing ELISA. For the ELISA, 125 μl of sample diluent from the kit was added to the tube, which was then briefly vortexed and 100 μl of the resulting mixture was added to an ELISA plate well. When all samples had been added to the ELISA plate, the plate was covered with an adhesive shield and incubated at 37°C for 30 min. After incubation, the plate was washed following the manufacturer's recommendations and blotted dry before 100 μl of conjugate solution from the kit was added to each well of the plate. The plate was then incubated for 30 min at 2–7°C, washed, and blotted dry. Then 100 μl of chromogen substrate solution from the kit was added to each well and the plate was incubated for 30 min at 18–30°C. Finally, 100 μl of a stop solution from the kit was added to each well and a microplate reader with a 450 nm filter was used to measure the optical density (OD) of each well. The interpretation of the results followed the National Veterinary Services Laboratories (NVSL)-SOP-0883 which states that a sample is non-negative if the OD is greater than the mean of 4 negative control wells plus 0.035. Samples with results situated just below the cut-off value (cut-off value-10%), as well as samples with an OD greater than or equal to the cut-off value were retested in duplicate, starting from the original homogenate. After repeating the ELISA, a sample was considered positive if at least 2 measurements were non-negative, and it was then submitted for further testing using IHC.

### Immunohistochemistry

All ELISA-positive and RT-QuIC-positive RLNs (*n* = 184) were tested by IHC at the MSU-VDL and by the NVSL. A sample was considered positive if IHC positive result was obtained from at least one of those laboratories. Another 188 randomly selected CWD negative RLN samples and prescapular lymph nodes, prefemoral lymph nodes, and popliteal lymph nodes (*n* = 22) were tested by IHC at the MSU-VDL. Formalin fixed lymph nodes were bisected, and tissue sections were incubated for 60 min with 95% formic acid followed by a 24-h rinse in tap water. Tissues were transferred into 10% neutral buffered formalin and after routine overnight deparaffinization, lymph node sections were embedded into paraffin blocks. Sections were cut at 5 to 7 μm thickness and slides were dewaxed, rehydrated, and treated in 95% formic acid for 5 min followed by multiple washes in Tris buffer at pH 7.5. Following antigen retrieval using the DAKO target retrieval solution (10X concentrate, S2369, Agilent, Santa Clara, CA) in a decloaker (BioCare Medical, Pacheco, CA), slides were immunohistochemically labeled for PrP^CWD^ following the NVSL protocol (SOP-PS-0002) (31, 32). Briefly, IHC was performed with the Ventana Discovery Ultra autostainer (Roche Diagnostics, Indianapolis, IN) that utilizes the kit-supplied F99 antibody (Anti-Prion Research Kit, RTU, Cat# 760-231; Roche Diagnostics, Indianapolis, IN), a biotinylated secondary antibody, an alkaline phosphatase-streptavidin conjugate, a substrate chromogen (fast red A, naphthol, fast red B), and hematoxylin counterstaining (Roche Diagnostics, Indianapolis, IN). A positive control section was included with every batch. The slides were examined according to the NVSL standards, single slides were analyzed and sections of examined lymph node included at least six follicles to allow for a diagnosis of negative in nodes without IHC labeling. Tissues were classified as positive if they had characteristic bright red, granular immunolabeling in the follicular centers.

### Real-Time Quaking-Induced Conversion Procedure

For RT-QuIC, RLNs were analyzed either unblinded (*n* = 698) or in a blinded manner (*n* = 602). Lymph nodes were trimmed and homogenized in a tube containing beads (ceramic bead and lysing matrix A, MP Biomedicals, Irvine, CA) and 1X PBS with 0.1% sodium dodecyl sulfate at 10% weight per volume using a FastPrep® (MP Biomedicals, Irvine, CA) bead-beater homogenizer (five cycles of 45 s at 6.5 m/s) with care to avoid any cross contamination among tissue homogenates. The homogenized samples were then immediately used or stored at−80°C until processed. RT-QuIC assays were performed as previously described (33) with a few modifications. Briefly, the RT-QuIC reactions were set up in 384-well clear bottom optic plates (Nunc) and consisted of 49 μl of RT-QuIC buffer (0.1 mg/ml truncated Syrian hamster recombinant prion protein (CWD Evolution, San Diego, CA), 320 mM NaCl, 20 mM Na_2_HPO_4_, 1mM EDTA, and 10 μM Thioflavin T) and 1 μl of sample. Positive control (ELISA, IHC and RT-QuIC positive sample), negative control (ELISA, IHC and RT-QuIC negative sample) and samples consisted of serial dilutions (10^−3^ and 10^−4^) of a 10% lymph node homogenate and were run in triplicate if 10^−3^ and 10^−4^ dilutions were used or quadruplicate if only dilution 10^−3^ was being tested. The 384-well plates were placed in a BMG Fluostar Omega plate reader with settings of 50 °C for 40 h with cycles of 1 min rest and 1 min shake (700 rpm, double orbital). Thioflavin T fluorescence measurements were taken every 15 min at a gain of 1,600 and excitation of 450 nm and emission of 480 nm. Data were processed using Mars Analytical Software (BMG Labtech, Cary, NC). Reactions were considered positive if fluorescence values exceeded the cycle threshold (average of the ten first readings plus 10 standard deviations). The time to threshold was defined as the time at which a sample fluorescence emission crossed the cycle threshold. Following the first analyses, every sample which showed partial positive reactions (1 to 3 out of 4 replicates) or did not have the same result as the ELISA test was reanalyzed. A sample was considered positive when at least half of total replicates surpassed the threshold.

### Statistical Analysis

Statistical analysis (mean, standard error of the mean (S.E.M.), *kappa* value and the percent agreement) were calculated using Microsoft Excel (Microsoft Inc.).

## Results

### RT-QuIC Results Are Comparable to ELISA and IHC

A comparison between currently recognized diagnostic methods for CWD, such as IHC and ELISA vs. new amplification assays, such as RT-QuIC is an important step to provide insight into the sensitivity and specificity of gold standard diagnosis and on the validation of new tools.

**Figure 2:**
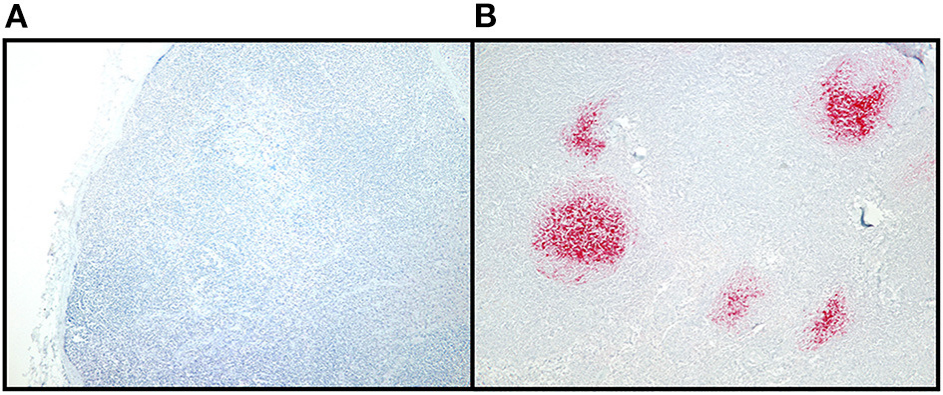
CWD detection in WTD RLN by IHC. Sections of examined lymph node included at least six follicles to allow for a diagnosis of negative in nodes without IHC labeling. Tissues were classified as positive if they had characteristic bright red, granular immunolabeling in the follicular centers. **(A)** IHC staining of a CWD negative RLN, at 10X magnification. **(B)** IHC staining of a positive RLN at 10X magnification.

Of the 1,300 RLNs tested, 184 (14.2%) were CWD positive by ELISA and 178 (13.7%) by RT-QuIC. Of the 184 ELISA and/or RT-QuIC positive samples, 176 were also IHC positive (Table 1). IHC positive lymph nodes presented characteristic granular immunolabelling in the follicular centers (bright red staining) (Figure 2). There was a total of seven samples out of the 1,300 RLNs tested (0.5%) where the results for IHC, ELISA, and RT-QuIC did not match. All seven samples were IHC negative and ELISA positive. Six out of the seven discordant samples presented negative RT-QuIC results, which matched the IHC outcomes. One sample was positive by RT-QuIC and ELISA and negative by IHC (Table 2). The percent agreement and *kappa* value between RT-QuIC and ELISA were 99.5 % and 0.98, respectively and between RT-QuIC and IHC were 99.7 % and 0.99, respectively. The percent agreement between ELISA and IHC was 98.1% and the *kappa* value 0.96.

**Table 1:** Number of positive, negative, not available (N/A), and total of RLNs tested by each assay.

	**Positive**	**Negative**	**N/A**	**Total**
**ELISA**	184	1,116	0	1,300
**RT-QuIC**	178	1,122	0	1,300
**IHC**	176	195	1	372^*^

*IHC was performed in all RT-QuIC-positive and ELISA-positive samples, except for one sample that we did not have enough tissue to be analyzed by IHC (N/A = 1), and 188 randomly selected negative RLNs. N/A, not available material to be analyzed*;

* *IHC was performed in RT/QuIC-positive and ELISA-positive samples (n = 184) and in 188 randomly selected negative samples*.

**Table 2:** Samples with discordant results between ELISA, IHC, and RT-QuIC.

**Sample ID**	**Sex**	**Age (yo)**	**Location**	**Average ELISA OD**	**ELISA result**	**IHC result**	**RT-QuIC result**
454295	M	1.5	Clinton	0.513	Positive	Negative	Negative
582500	F	1	Macomb	0.167	Positive	Negative	Negative
583708	F	0	Mecosta	0.644	Positive	Negative	Negative
576488	F	4	Macomb	0.211	Positive	Negative	Negative
685995	F	2	Washtenaw	0.261	Positive	Negative	Negative
521225	M	1	Clinton	0.065	Positive	Negative	Negative
575795	M	1	Macomb	3.328	Positive	Negative	Positive

*ELISA positive, sample with average value > 0.035 plus the average of the negative control. IHC was performed at the MSU-VDL and NVSL and a sample was considered IHC positive if a positive result was reported by at least one of those laboratories. RT-QuIC threshold was calculated by the average fluorescence of the ten first readings plus 10 standard deviations. A sample was considered RT-QuIC positive when at least half of the total replicates surpassed the threshold. Yo, years old; OD, optical density*.

### Characteristics of the Positive CWD Cases

**Figure 3:**
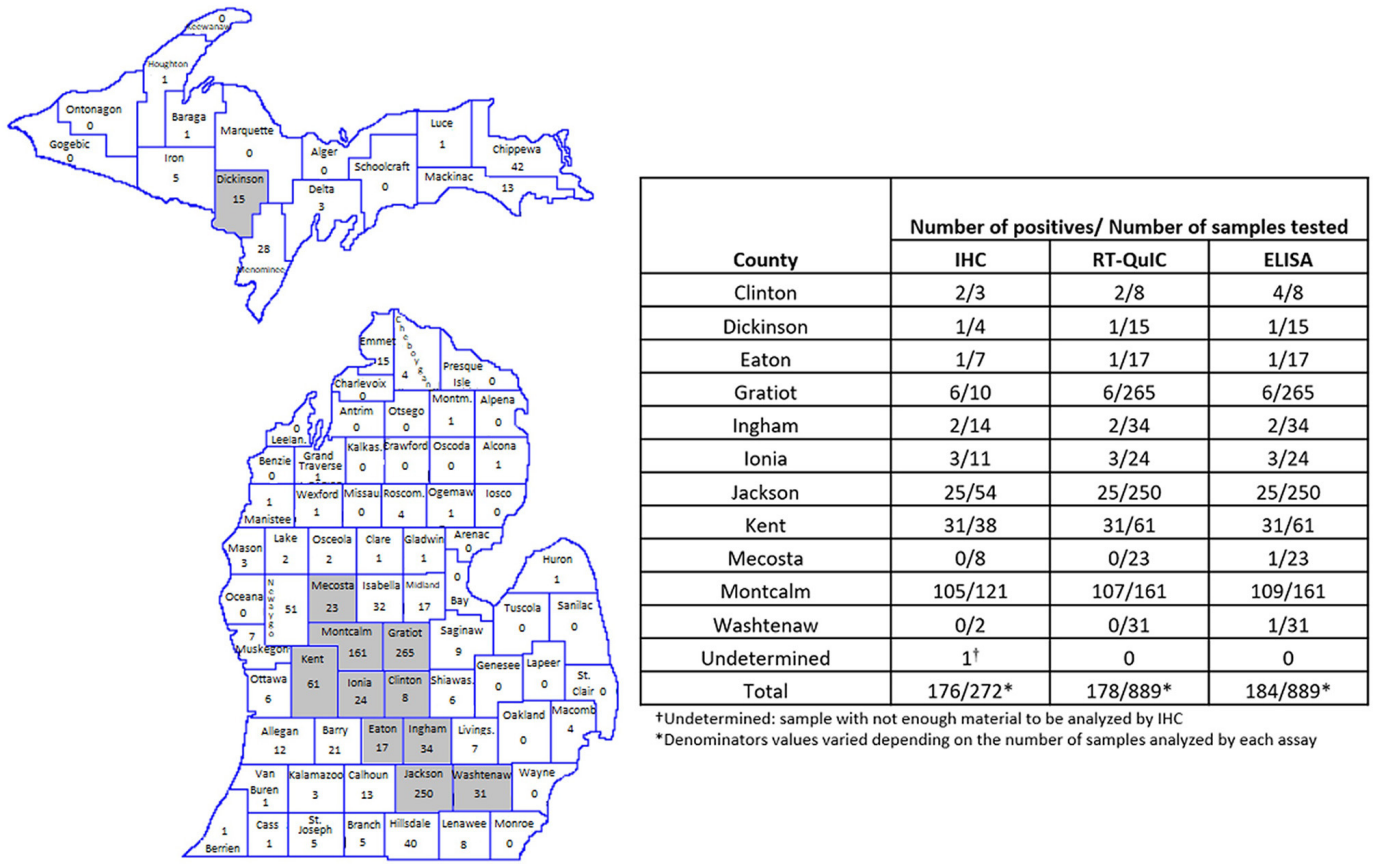
Amount and location of positive samples in Michigan. Map shows the total number of RLN samples tested in each County. Gray represents the Counties with CWD positive results. The table shows the number of positive samples/number of samples tested by County and by each technique used. RT-QuIC and ELISA were performed in 1,300 WTD RLNs. IHC was performed in all RT/QuIC-positive and ELISA-positive samples (*n* = 184) and in 188 randomly selected negative samples.

All, except one CWD positive RLNs analyzed were from ten Counties geographically located in the West Michigan region of the Lower Peninsula (Figure 3). Montcalm County alone accounted for more than half of the total of positive cases detected in this work (between 109 and 107 positives depending on the test performed). The other positive samples were concentrated in surrounding Counties: Clinton (2 positives by RT-QuIC and IHC and 4 by ELISA), Eaton (1 positive by all three assays), Gratiot (6 positives by all three assays), Ingham (2 positives by all three assays), Ionia (3 positives by all three assays), Jackson (25 positives by all three assays), Kent (31 positives by all three assays), Mecosta (1 positive by ELISA), Washtenaw (1 positive by ELISA) (Figure 3). One positive sample (by all three assays) was originated from Dickinson County in the Michigan Upper Peninsula (Figure 3).

Of the 1,300 samples analyzed, 20.2% of males were CWD positive in contrast with 9.9% of the females (Table 3). More than 3 years old (yo) to 4 yo animals had the highest percentage of positive cases (21.7%), followed by more than 1 to 2 yo animals (20.1%). The percentage of more than 2 to 3 yo positive CWD deer was 16.4%. The percentage of CWD positive cases was smaller in fawns (from 0 to 1 yo animals) (7.8%) and in older animals (more than 4 to 5 yo with 7% and WTD more than 5 yo with 5.8%) (Table 4).

**Table 3:** Positive PrP^CWD^ RLNs by sex.

	Total of positive (% of positive)^*^			**Total of samples tested**		
**Sex**	IHC^*^	**RT-QuIC**	**ELISA**	**IHC**	**RT-QuIC**	**ELISA**
Female	80	81 (9.9%)	85 (10.4%)	154	819	819
Male	95	96 (20.4%)	98 (20.8%)	216	470	470
Unknown	1	1 (9.1%)	1 (9.1%)	2	11	11
Total	176	178 (13.7)	184 (14.1%)	372	1,300	1,300

*The table presents the total and percentage of positive RLNs and the total of samples tested by sex and assay performed. It is important to note that the percentage of positive samples by sex was not calculated for IHC since only RT/QuIC-positive and ELISA-positive samples (n = 184) and 188 randomly selected RLNs were tested by this assay*.

* *Percentage of positive samples by sex was not calculated for IHC since the assay was only performed in RT/QuIC-positive and ELISA-positive samples (n = 184) and 188 randomly selected negative samples*.

**Table 4:** Positive PrP^CWD^ RLNs by age category.

	Total of positive (% of positive)^*^			**Total of samples tested**		
**Years old**	IHC^*^	**RT-QuIC**	**ELISA**	**IHC**	**RT-QuIC**	**ELISA**
0 to 1	38	40 (8.0%)	43 (8.6%)	118	501	501
> 1 to 2	68	68 (20.1%)	70 (20.6%)	125	339	339
> 2 to 3	37	37 (16.4%)	37 (16.4%)	73	225	225
> 3 to 4	26	26 (21.7%)	27 (22.5%)	38	120	120
> 4 to 5	4	4 (7.0%)	4 (7.0%)	9	57	57
> 5	3	3 (5.8%)	3 (5.8%)	6	52	52
Unknown	0	0	0	3	6	6
Total	176	178 (13.7%)	184 (14.1%)	372	1,300	1,300

*The table presents the total and percentage of positive RLNs and the total of samples tested by age category and assay performed. Age classes were determined based on tooth wear by experienced staff at the Michigan Department of Natural Resources-Wildlife Disease Laboratory. It is important to note that the percentage of positive samples by age category was not calculated for IHC since only RT/QuIC-positive and ELISA-positive samples (n = 184) and 188 randomly selected negative RLNs were tested by this assay*.

* *Percentage of positive samples by age category was not calculated for IHC since the assay was only performed in RT/QuIC-positive and ELISA-positive samples (n = 184) and 188 randomly selected negative samples*.

### PrP^CWD^ Detected in Prefemoral, Prescapular and Popliteal Deer Lymph Nodes

RLNs play an important role as a portal of entry for CWD in early accumulation and propagation of PrP^CWD^ in deer (34). Other early sites of prion accumulation are tonsils, Peyer's patches, and ileocaecal lymph nodes, followed by progressive involvement of central and peripheral nervous tissues (34, 35). However, the exact progression of the PrP^CWD^ deposition in different anatomic locations and the disease stage at which it occurs in peripheral lymph nodes and surrounding tissues is not well understood. This is especially important, since, even if there is no published evidence for CWD transmission to human-like primates at this time, the CWD species barrier between cervids and humans may not be fixed (36, 37). The risk for interspecies transmission through the consumption of CWD-contaminated meat may increase as CWD continues to spread and adapt (38).

A set of RLNs, prescapular lymph nodes, prefemoral lymph nodes and popliteal lymph nodes (Figure 1) from eight WTD were tested by ELISA, IHC and RT-QuIC. Animals that presented positive CWD results in the RLNs by ELISA, IHC, and RT-QuIC (*n* = 7), also presented PrP^CWD^ in all the other lymph nodes when tested by IHC and RT-QuIC (Table 5). The ELISA, on the other hand, did not detect PrP^CWD^ in prefemoral and popliteal lymph nodes from one positive deer (Table 5), but the results of prescapular lymph node and RLN from the same animal tested positive. All lymph nodes tested from CWD-negative control deer were negative by ELISA, IHC, and RT-QuIC.

**Table 5:** PrP^CWD^ detection on RLNs, prescapular lymph nodes, prefemoral lymph nodes, and popliteal lymph nodes of WTD by ELISA, IHC, and RT-QuIC.

					**Retropharyngeal LN**				**Prescapular LN**					**Prefemoral LN**					**Popliteal LN**				
**Sample**	**Sex**	**Age**	**Location**	**ELISA Avg. OD**	**ELISA Result**	**IHC Result**	**RT-QuIC Result**	**RT-QuIC Avg. time to Threshold**	**ELISA Avg. OD**	**ELISA Result**	**IHC Result**	**RT-QuIC Result**	**RT-QuIC Avg. time to Threshold**	**ELISA Avg. OD**	**ELISA Result**	**IHC Result**	**RT-QuIC Result**	**RT-QuIC Avg. time to Threshold**	**ELISA Avg. OD**	**ELISA Result**	**IHC Result**	**RT-QuIC Result**	**RT-QuIC Avg. time to Threshold**
702671	M	1.5	Jackson	3.08	positive	positive	positive	10.31	1.275	positive	positive	positive	18.12	N/A	N/A	positive	positive	8.87	0.696	positive	positive	positive	15.4
702880	F	0	Jackson	4.14	positive	positive	positive	8.12	3.555	positive	positive	positive	8.73	4.118	positive	positive	positive	7.23	3.914	positive	positive	positive	7.93
675730	F	4	Jackson	2.24	positive	positive	positive	7.56	2.502	positive	positive	positive	6.13	N/A	N/A	N/A	N/A	N/A	2.29	positive	positive	positive	8.07
674269	F	2	Jackson	2.94	positive	positive	positive	6.01	4.105	positive	positive	positive	7.2	N/A	N/A	N/A	N/A	N/A	N/A	N/A	positive	positive	6.84
674291	F	5	Jackson	3.48	positive	positive	positive	6.56	4.125	positive	positive	positive	6.04	4.044	positive	positive	positive	6.71	4.154	positive	positive	positive	5.83
674292	F	0	Jackson	0.24	positive	positive	positive	7.03	0.227	positive	positive	positive	6.3	0.008	negative	positive	positive	11.68	0.01	negative	positive	positive	17.56
674780	F	3	Jackson	3.782	positive	positive	positive	9.13	1.766	positive	positive	positive	11.3	3.368	positive	positive	positive	11.15	4.079	positive	positive	positive	10.65
420830	F	12	Hillsdale	0.011	negative	negative	negative	N/A	0.007	negative	negative	negative	N/A	0.01	negative	negative	negative	N/A	0.01	negative	negative	negative	N/A

*ELISA positive, sample with average value > 0.035 plus the average of the negative control. RT-QuIC threshold was calculated by the average fluorescence of the ten first readings plus 10 standard deviations. The time to threshold was defined as the time at which a sample fluorescence emission crossed the cycle threshold. A sample was considered RT-QuIC positive when at least half of the total replicates surpassed the threshold. N/A, not available*.

## Discussion

CWD was first identified more than 50 years ago in a captive mule deer at Colorado State University (Fort Collins, Colorado). Since its discovery, the disease has been reported across North America, in South Korea, and in Europe (1–5). In Michigan, CWD was first described in a deer farm in Kent County in 2008. Since 2015, when the first free-ranging CWD-positive deer was identified in Michigan, the disease has been reported in several counties in the Lower Peninsula. In October 2018, the first CWD-positive deer was found in the Upper Peninsula in Dickinson County (Figure 3)^1^ (39). As the endemic areas have expanded, so has the need for rapid, sensitive, and cost-effective diagnostic tests. The current gold standard for confirmatory CWD testing in WTD is IHC of the medial RLN; however, ELISA is a high-throughput test often used to screen samples prior to IHC (31, 40). These antibody-based prion detection tests have been very helpful in providing an understanding of pathogenesis, transmission, and geographic distribution of CWD and other prion diseases over the past few decades. However, there are many concerns about their sensitivity especially for animals in early stages of the disease to be effective in robust surveillance programs (41–43). The development of *in vitro* amplification assays, as protein misfolding cyclic amplification and RT-QuIC have helped to enhance the sensitivity of CWD prion detection in the last few years. The RT-QuIC assay has been reported to amplify PrP^CWD^ seeds present in brain dilutions in the femtogram range (44, 45) and has been used to detect PrP^CWD^ in a variety of anatomic sites, body fluids, and excreta (13, 22–30), but the technique has yet to be approved for official CWD surveillance.

In this work, we first evaluated the efficacy of the RT-QuIC assay in detecting CWD prions in RLNs of WTD and compared with the results from IHC and ELISA. We found that our RT-QuIC results were comparable to those of the IHC and ELISA, with only 0.5% (7 samples out of 1,300) of discordant results between the three techniques when testing WTD RLNs (Table 2). In previous studies that compared the three assays, 100% of matching results were observed (23, 30). One possible explanation for the observed difference is the fact that the previous studies did not include as many positive WTD RLNs. All the discordant samples in our study were IHC negative but positive by ELISA (*n* = 7). A previous study comparing ELISA and IHC results also detected inconsistent results between the two techniques in 12 out of 101 (11.9%) RLNs tested (46). The same study demonstrated the importance of testing contralateral RLNs by IHC since PrP^CWD^ may not be bilaterally present in those tissues (46). Our failure to confirm ELISA diagnoses with IHC and RT-QuIC are likely either due to ELISA results in discrepant samples were false positives, especially on the samples with low OD ELISA value (*n* = 6) where IHC and RT-QuIC presented matching results (Table 2) or due to the sampling methods and protein distribution within the RLN, since the practice of using 1 section of lymph node per animal for IHC may result in false-negative results, especially in early-infection cases (41). RT-QuIC assay only requires sampling between 70 and 100 mg compared with 180–220 mg needed for ELISA, which are preferentially collected from the lymph node cortex to improve accuracy. Nevertheless, one potential caveat of both ELISA and RT-QuIC is that it is likely not possible to be certain that the correct part of the tissue sample was collected and tested. Whereas, IHC examines cross sections of RLN that are 5–7 μm in thickness and include both cortex and medulla. Moreover, the sections collected for IHC contain a fraction of the tissue sampled by ELISA and RT-QuIC. Therefore, it should be expected that several sections sometimes must be prepared for IHC before positive follicles are observed in early CWD cases (41). Ultimately, we observed that all 176 samples that were positive by IHC were also positive by RT-QuIC. Conversely, no IHC-positive/RT-QuIC-negative samples were identified (Table 2). Only one of our discordant results was a RT-QuIC-positive sample (also ELISA-positive) that was negative by IHC at MSU-VDL and NVSL. Previous studies have shown that RT-QuIC positivity precedes and ultimately correlates with PrP^CWD^ deposition observed by IHC, reflecting greater sensitivity of RT-QuIC vs. IHC especially for early CWD detection (47, 48). However, our sample is most likely from an animal with established CWD disease, since it presented a high mean OD value (3.328) on ELISA (Table 2). The same was observed in another study comparing ELISA and IHC, where a sample with a high OD value was negative by IHC (41). The result may be explained by differences in methodologies or affinity between antiPrP antibody used in the ELISA, recombinant prion protein used in the RT-QuIC, and the monoclonal antibody used for IHC. Another explanation would be differences in the spatial distribution of the PrP^CWD^ in the samples tested by each assay. Taken together, our results show a great correlation of results between the three techniques, with an agreement between RT-QuIC and the other assays > 99.5% and *kappa* value > 0.98, which was greater than the agreement and *kappa* value observed between IHC vs. ELISA (98.1% and 0.96, respectively). Since the spatial distribution of prion protein within the RLN may play a role in whether protein is detected (41), to achieve a higher confidence in test results, it seems prudent to examine multiple sections of the RLNs, as well as both RLNs to confirm or refute CWD infection. Furthermore, more studies are needed to evaluate the efficiency of different recombinant prion protein in samples from different CWD strains.

Similar to what has been reported for scrapie in sheep (49), PrP^CWD^ spreads throughout the body following a general pattern of prion accumulation, characterized by relatively rapid and widespread prion accumulation in the lymphatic tissues, followed by progressive involvement of and lesions in central and peripheral nervous tissues (34, 35, 50). Lastly, involvement of a wider variety of tissues and organs throughout the body, including endocrine system, heart, kidney, lung, and muscle tissues can be observed, albeit in small amounts, as animals become terminally ill (29, 35). Nevertheless, the exact progression of the PrP^CWD^ deposition and what time in the disease progression it happens in peripheral tissues is unclear. Mule deer fawns inoculated orally with CWD showed early accumulation of PrP^CWD^ in Peyer's patches and ileocecal lymph nodes, as well as the RLNs, as early as 42 days post infection (34). However, the proportion of positive follicles was higher in RLNs than in Peyer's patches and ileocecal lymph nodes from 42 to 80 days post infection (34), suggesting that RLNs may play an important role as a portal of entry for CWD in early propagation and accumulation of PrP^CWD^ in deer. Another study observed PrP^CWD^ deposits in tonsils and lymph nodes prior to detectable accumulation in intestinal lymphoid aggregates at 90 days post infection (35). Furthermore, a study shown that peripheral accumulation and the excretion of the infective prion protein can occur at the same time as the peripheral lymphoid accumulation (51). Finally, in very early cases of CWD, PrP^CWD^ deposition is limited and since RLNs and medulla oblongata at the level of the obex are early sites of prion accumulation, they are considered gold standard tissues for post-mortem CWD detection (35).

In this study, in addition to analyzing the performance of the RT-QuIC assay compared with ELISA and IHC in RLNs, we also tested a broad set of WTD lymph nodes from different parts of the body (Figure 1). We found that in addition to the RLN, PrP^CWD^ was also present in prescapular lymph nodes, prefemoral lymph nodes, and popliteal lymph nodes of all CWD positive WTD tested here (Table 5). Our results are concordant with previous work reporting that CWD prions are distributed across lymphoid tissues from deer orally infected with CWD (34, 35, 52). RT-QuIC and ELISA were able to detect PrP^CWD^ in all the lymph nodes tested from the CWD-positive animals. On the other hand, ELISA was negative in samples from prefemoral lymph node and popliteal lymph node from one of the CWD-positive WTD. The same animal presented positive ELISA results when RLN and prescapular lymph nodes were tested (Table 5). It is interesting to note that this animal presented low OD ELISA values of 0.24 and 0.227, for RLN and prescapular lymph node, respectively (plate-to-plate cut-off ELISA OD values ranged from 0.038 and 0.08, data not shown), which is indicative of early CWD infection. It is known that after entering the body, either orally and/or intranasally, CWD prions typically accumulate within the lymphatic tissues of the head and neck, such as the RLNs, before spreading from the head/neck lymphoid tissues to the rest of the body (34, 35, 50). The RT-QuIC results for the same animal also suggest low accumulation of PrP^CWD^ in the prefemoral lymph node and popliteal lymph node, which were detected positive after 11 and 17 h, respectively, compared to 7 h for RLN and 6 h for prescapular lymph node. Our results suggest that RT-QuIC may have been more sensitive than ELISA to detect PrP^CWD^ accumulation in lymph nodes other than the RLN in animals in early stages of the disease. Although additional research, including more lymphoid tissues from different parts of the body, is needed to determine the full extent to which CWD prions occur within specific lymph nodes and surrounding tissues of infected animals.

Deer behave differently among sex and age classes, and as a result, CWD prevalence can differ among different sex and age groups. Most likely, the sex and age effects are mainly driven by differences in pathogen exposure (53) and therefore, strongly depend on the social organization or behavior of a given species. In our study, we observed a two-fold higher CWD prevalence in males (20.2%) than in females (9.9%) WTD (Table 3). These results are in accordance with previous work in both mule deer and WTD (54–56). Female deer are known to form matrilineal groups with stable home ranges where they consistently interact with the same members of their matrilineal groups (54, 57). On the other hand, males are hypothesized to have higher CWD prevalence due to behavioral traits related to serial polygamy, and longer movement distances, increasing the overall likelihood of visiting an infected group or spreading the disease to new groups (57, 58). On the other hand, a previous study observed higher CWD prevalence in females when compared with males, suggesting that in some more heavily CWD affected areas, environmental transmission in late epidemic stages may erode the sex-specific infections in more concentrated populations (59). As previously reported (54, 56, 60), we also found that CWD prevalence is very low in fawns (7.8% CWD prevalence in WTD <1 year old) when compared with other age groups. This observation can be explained by the pattern of infection across age classes. CWD likely arises due to differences in the prion exposure period since birth and the long incubation period of the infection before it can be detected by standard diagnostic tests (61). We observed that CWD prevalence increases in yearlings (between 1 and 2 yo; 20.1%), declining slightly in the third year of life (between 2 and 3 yo; 16.4%), and returning to increase with a peak in the fourth year of life (between 3 and 4 yo; 21.7%) (Table 4). After the fourth year of life (more than 4 yo), CWD prevalence declines steadily. In agreement with our results, it has been previously reported that prevalence levels often continue to increase moderately with age in the adult stage, where greater movement and interactive behaviors lead to higher infection risk (60, 62). Furthermore, a decline in CWD infection among the oldest deer has also been previously reported in both WTD and mule deer (59, 62) that can result from decreased movement and interaction behaviors leading to a lower force of infection and disease-induced mortality removing older individuals from the population.

Taken together, we show evidence that the RT-QuIC assay is comparable to ELISA and IHC for CWD detection and could be used as a new tool for the detection of CWD prions. In this study, RT-QuIC and IHC (which is the gold standard technique for CWD diagnostic) results showed better correlation than with ELISA. One of the advantages of the RT-QuIC is that it can easily scaled up when compared to IHC and ELISA, since it can be performed in 384-well plates, which is amenable to CWD surveillance programs across the globe. Another advantage of the RT-QuIC is that the final product is not infectious. When compared to IHC, RT-QuIC is less expensive and less laborious. RT-QuIC can also portends IHC positivity in signifying early CWD infection before it can be detected by IHC (47, 48). Moreover, RT-QuIC results can be visualized while the assay is running, and a positive sample can be detected as soon as the fluorescence values start to increase. Furthermore, RT-QuIC can also be used in a variety of tissues, body fluids, and excreta from animals at clinical, and sometimes at subclinical stages of the disease (13, 22–29). Finally, it can also provide a range of information that may be used for the assessment of tissue burden and environmental contamination, which will allow for more detailed studies into disease epidemiology and pathogenesis (25, 63, 64). Our findings also suggest that CWD prions occur throughout an array of WTD lymph nodes, but further studies focusing on larger sample sizes are necessary to understand the extent of this distribution not only in lymphoid tissues, but also in surrounding organs and tissues. Continued improvements in RT-QuIC methodologies, especially for antemortem CWD diagnosis and multi-laboratory validation on blinded sample panels are ongoing and will aid in defining an algorithm for RT-QuIC application in prion diagnostics and routine CWD surveillance.

## Data Availability Statement

The original contributions presented in the study are included in the article/supplementary material, further inquiries can be directed to the corresponding author/s.

## Author Contributions

CH, KS, SB, and SS designed the study. CH, JD, NG, and MK performed the experiments. CH and JD analyzed the data. CH and SS wrote the manuscript. All authors read and approved the final manuscript.

## Funding

The research was supported by the Department of Natural Resources, Grant No. CWD20-004.

## Conflict of Interest

The authors declare that the research was conducted in the absence of any commercial or financial relationships that could be construed as a potential conflict of interest.

## Publisher's Note

All claims expressed in this article are solely those of the authors and do not necessarily represent those of their affiliated organizations, or those of the publisher, the editors and the reviewers. Any product that may be evaluated in this article, or claim that may be made by its manufacturer, is not guaranteed or endorsed by the publisher.

## Footnotes

^1^ https://www.michigan.gov/dnr

## Abbreviations

CWD: Chronic wasting disease
IHC: Immunohistochemistry
MSU-VDL, Michigan State University: Veterinary Diagnostic Laboratory
NVSL: National Veterinary Services Laboratories
OD: optical density
PrP^CWD^: misfolded prion protein
RLN: retropharyngeal lymph node
RT-QuIC: Real-time Quaking-Induced Conversion
WTD: White-tailed deer
yo: years old.
